# Case report: Virtual reality-based arm and leg cycling combined with transcutaneous electrical spinal cord stimulation for early treatment of a cervical spinal cord injured patient

**DOI:** 10.3389/fnins.2024.1380467

**Published:** 2024-05-17

**Authors:** Xiaolei Chu, Shuaiyi Liu, Xiaoxuan Zhao, Tao Liu, Zheng Xing, Qingwen Li, Qi Li

**Affiliations:** ^1^Department of Rehabilitation, Tianjin University Tianjin Hospital, Tianjin, China; ^2^Tianjin Key Laboratory of Exercise Physiology and Sports Medicine, Institute of Sport, Exercise and Health, Tianjin University of Sport, Tianjin, China

**Keywords:** spinal cord injury, virtual reality, cycling, electrical stimulation, early treatment

## Abstract

Spinal cord injury is a condition affecting the central nervous system, causing different levels of dysfunction below the point of nerve damage. A 50-year-old woman suffered a neck injury as a result of a car accident. After undergoing posterior cervical C3–C6 internal fixation with titanium plates on one side and C7 lamina decompression, the patient, who had been diagnosed with C3–C7 cervical disk herniation and spinal stenosis causing persistent compression of the spinal cord, was transferred to the rehabilitation department. After implementing the combined therapy of Virtual Reality-based arm and leg cycling along with transcutaneous electrical stimulation of the spinal cord, the patients experienced a notable enhancement in both sensory and motor abilities as per the ASIA scores. The patient’s anxiety and depression were reduced as measured by the Hamilton Anxiety and Hamilton Depression Tests. As evaluated by the SCIM-III, the patient’s self-reliance and capacity to carry out everyday tasks showed ongoing enhancement, leading to the restoration of their functionality. Hence, the use of Virtual Reality-based arm and leg cycling along with transcutaneous electrical spinal cord stimulation has potential to positively impact function in patients with spinal cord injury. However, as this is a case report, the small number of patients and the fact that the intervention was initiated early after the injury, we were unable to separate the recovery due to the intervention from the natural recovery that is known to occur in the initial weeks and months after SCI. Therefore, further randomized controlled trials with a large sample size is necessary.

## 1 Introduction

Spinal cord injury (SCI) is known to induce a loss of motor and sensory function, often resulting from traffic accidents or falls from significant heights, thereby inflicting severe damage upon the central nervous system. This condition is widely recognized as a profoundly detrimental threat to human welfare, with its incidence steadily increasing on a global scale (Fehlings et al., 2017). The rehabilitation of SCI has historically presented a considerable obstacle within clinical settings. In contrast to the peripheral nervous system (PNS), the central nervous system (CNS) exhibits restricted regenerative capabilities. Despite notable advancements in surgical techniques and pharmaceutical interventions, a lack of efficacious therapeutic alternatives persists in terms of fundamentally enhancing functional recuperation. Moreover, SCI not only inflicts severe physical and psychological trauma upon patients (Majdan et al., 2017), but also imposes a substantial economic burden on both society and families due to suboptimal rehabilitation outcomes and excessive reliance on nursing care (Ge et al., 2018). As a medical condition, SCI is distinguished by its elevated prevalence (Fehlings et al., 2017), significant disability (Anderson, 2004; Fawcett et al., 2007), and heightened mortality rates (Song et al., 2022).

Arm and Leg Cycling is a mode of training in which the patients’ limbs are coordinated at the same time through patient’s active force application or a motor-driven linkage device. Studies have shown that this exercise modality has been widely used for functional recovery in SCI patients and has shown good efficacy in promoting improvement in motor function after SCI (Mac-Thiong et al., 2021). Virtual reality technology is an emerging comprehensive technology that integrates various technologies such as sensing technology, human interaction technology and simulation technology (Schiza et al., 2019). Virtual Reality-based rehabilitation has been shown to improve SCI patients’ motor dysfunction and promote higher levels of brain activation (Lim et al., 2020) and quality of life (Zanatta et al., 2023). Transcutaneous electrical spinal cord stimulation (tSCS), a noninvasive electrical stimulation modality that places electrodes on the surface of the skin and stimulates spinal cord circuits with electrical current, has been shown to activate neural circuits by recruiting afferent fibers located in the dorsal roots and increasing the excitability of spinal cord circuits (Gerasimenko et al., 2015). All three therapies may promote recovery from spinal cord injury, but their combined effect is unknown to us.

Besides, the selection of treatment modality and duration holds significant importance, thus prompting our research efforts to explore the therapeutic effects on patients with SCI through a comprehensive examination of the perspectives of the brain, spinal cord, and peripheral system. This study examines the effectiveness of combining Virtual Reality-based arm and leg cycling with tSCS for early treatment of patients with cervical SCI.

## 2 Case description

### 2.1 History

**Figure 1:**
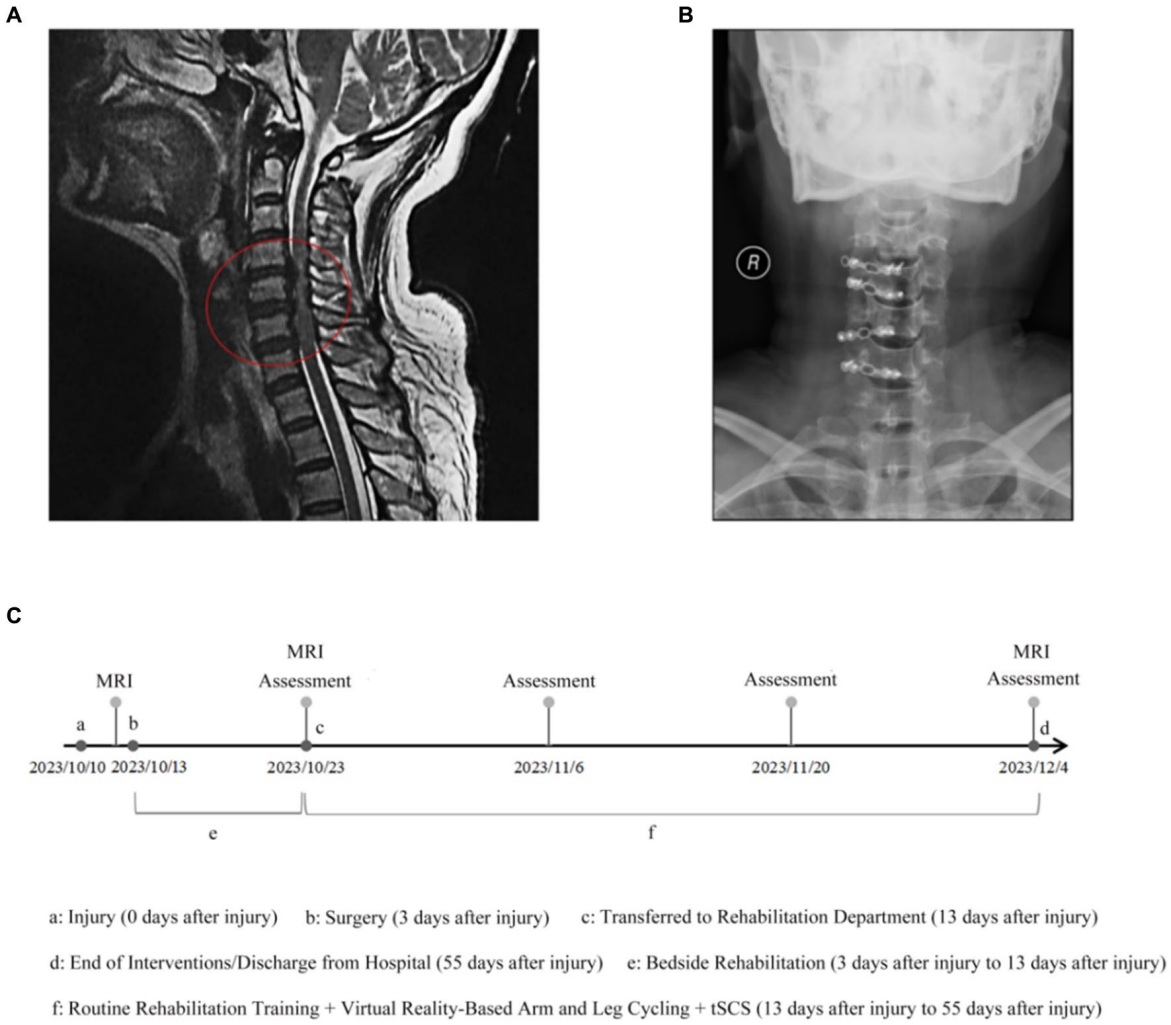
MRI images and Radiographic before treatment. **(A)** Cervical magnetic resonance imaging showed C3–C7 cervical disk herniation associated with spinal stenosis that cause the spinal cord persistent oppression. **(B)** Postoperative radiograph. **(C)** Rehabilitation timeline chart. Assessments include ASIA, HAMA, HAMD, and SCIM-III.

In Oct 2023, a 50-year-old woman was admitted to our hospital with a SCI caused by a car accident. She experienced neck pain, numbness, and weakness in her extremities. After admission, she was diagnosed with spinal stenosis and C3–C7 cervical disk herniation, which caused constant pressure on her spinal cord (Figure 1A). The final determination was incomplete quadriplegia resulting from cervical SCI, according to the American Spinal Injury Association impairment scale D. A comprehensive assessment involving laboratory tests, physical examination, and imaging did not identify any evident contraindications. The posterior cervical C3-C6 region was internally stabilized with titanium plates for the unilateral open door approach, while lamina decompression was performed at the cervical C7 level (Figure 1B).

Within 10 days of surgery, the patient underwent bedside rehabilitation in the spine surgery department, including passive joint mobilization, sensory stimulation, and respiratory training for 30 min once a day for 10 days (Supplementary Table S1). Due to the patient’s long recovery time from her cervical spinal cord injury, she needed a more effective treatment program. Therefore, she decided to be transferred to the rehabilitation department for long-term rehabilitation interventions, including respiratory training, neuromuscular electrical stimulation and limb integration training. In addition, in order to further optimize the therapeutic effects, the patient decided to undergo a combination therapy of Virtual Reality arm and leg cycling exercises combined with tSCS (Figure 1C).

### 2.2 Diagnosis

Following her initial appointment, the patient underwent a comprehensive clinical assessment, during which no discernible abnormalities were detected in the joints of her upper and lower extremities. In accordance with ASIA and ISCoS International Standards Committee (2019) International Standards for Neurological Classification of Spinal Cord Injury (ISNCSCI), her neurological injury level was determined to be C4, with an AIS D rating. The individual exhibited heightened sensitivity to gentle touch and needle-like sensations in her shoulders, upper limbs, and hands. Hypoaesthesia, characterized by reduced perception of light touch and pin prick sensations, was observed in the trunk below the T4 dermatomes and in the lower limbs. The motor strength grade for the elbow flexor, wrist extensor, elbow extensor, hip flexor, knee extensor, ankle dorsi flexor, digitorum longus extensor, and ankle plantar flexor was quantified as 3 points through muscle testing. Additionally, the left middle finger flexor and little finger abduction exhibited a motor strength grade of 2 points. The visibility or detectability of the flexor muscle of the middle finger and the abduction of the little finger were absent. The individual exhibited limited capacity to perform activities of daily living (ADL) and required substantial assistance. Consequently, a combination therapy approach was selected. The patient granted consent for the publication of this case report.

## 3 Intervention and outcome

The proposed intervention involved the utilization of Virtual Reality-based arm and leg cycling, which integrated multiple stimuli and feedback from the exercising limbs. This approach aimed to enhance sensory and locomotor functional recovery by activating both the cerebral and limb regions. Furthermore, the concurrent administration of tSCS to both the cervical and lumbosacral regions has the potential to induce a stimulus interaction that alters the excitability of multiple segments within the spinal cord (Barss et al., 2022).

### 3.1 Virtual reality-based arm and leg cycling

**Figure 2:**
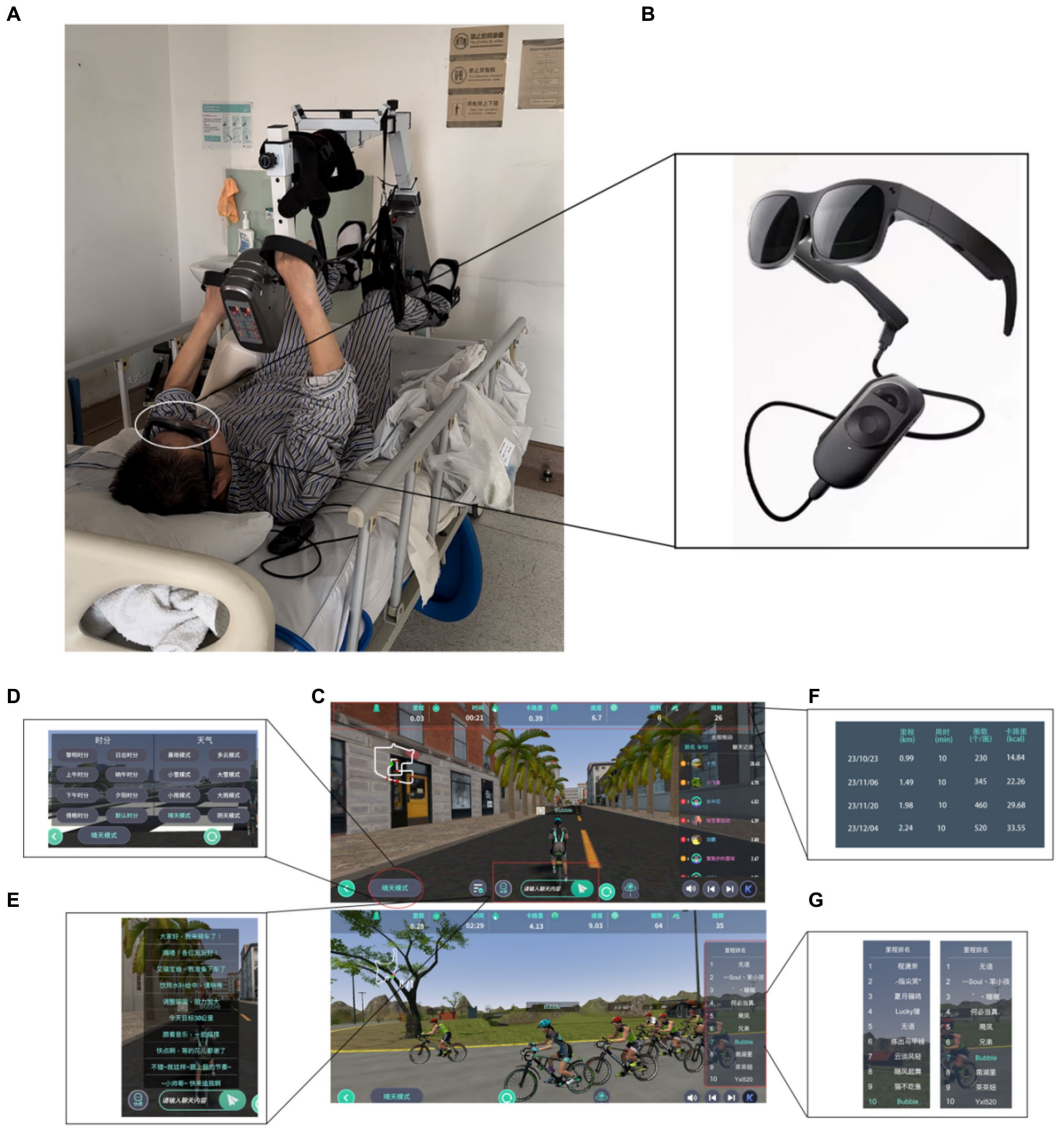
Patient undergoing Virtual Reality-based arm and leg cycling in therapy. **(A)** Real-world scenario: Virtual Reality-based limb-linkage rehabilitation training. **(B)** Virtual Reality glasses. **(C)** Virtual Scenario: Bicycle Race. **(D)** Virtual Scenario: time and weather. **(E)** Live chat in cycling. **(F)** Self-exercise goals for patient at different stages. **(G)** Real-time mileage ranking during the campaign. The above C-G images are from version 3.3.2 of the Dong Qi APP by Jinhua Zhanyue Intelligent Technology Company, China.

Virtual reality-based arm and leg cycling started on the same day after completion of routine rehabilitation training (Figure 2A). A crucial aspect of Virtual Reality-based rehabilitation for individuals with SCI was the alignment between the virtual environment and the corresponding physical movements. Consequently, the arm and leg cycling device (DN-813, China) was linked to the VR glasses (Lei Niao Air Plus, Huizhou TCL Mobile Communication Company, China) through a sensor connector (Figures 2B,C). The patient actively operated the device to facilitate synchronized movements of the upper and lower limbs, while character engaged in cycling activities on the simulated road (Figure 2D). There are several aspects to elucidate. Firstly, the synchronization between the sensor connection and the character’s pedaling movements, lap count, and frequency, as presented in the glasses, is noteworthy. Secondly, the therapist possesses the ability to independently regulate alterations in the virtual environment, including variations in time of day (sunrise, noon, and sunset) and weather conditions (sunny, cloudy, and snowy), which simulate changes in the cycling environment (Figure 2E). Additionally, the incorporation of supplementary virtual participants during the ride establishes a competitive environment, prompting the patient to engage in more extensive physical activity (Figure 2G). Moreover, the virtual image’s upper column offers instantaneous feedback on the patient’s exercise metrics, encompassing distance covered, duration, calories burned, and laps completed. Patients are empowered to establish daily exercise objectives for themselves (Figure 2F) by leveraging the real-time feedback, individuals can strive toward achieving their exercise goals and ensure the fulfillment of their daily exercise routine.

### 3.2 Transcutaneous electrical spinal cord stimulation

**Figure 3:**
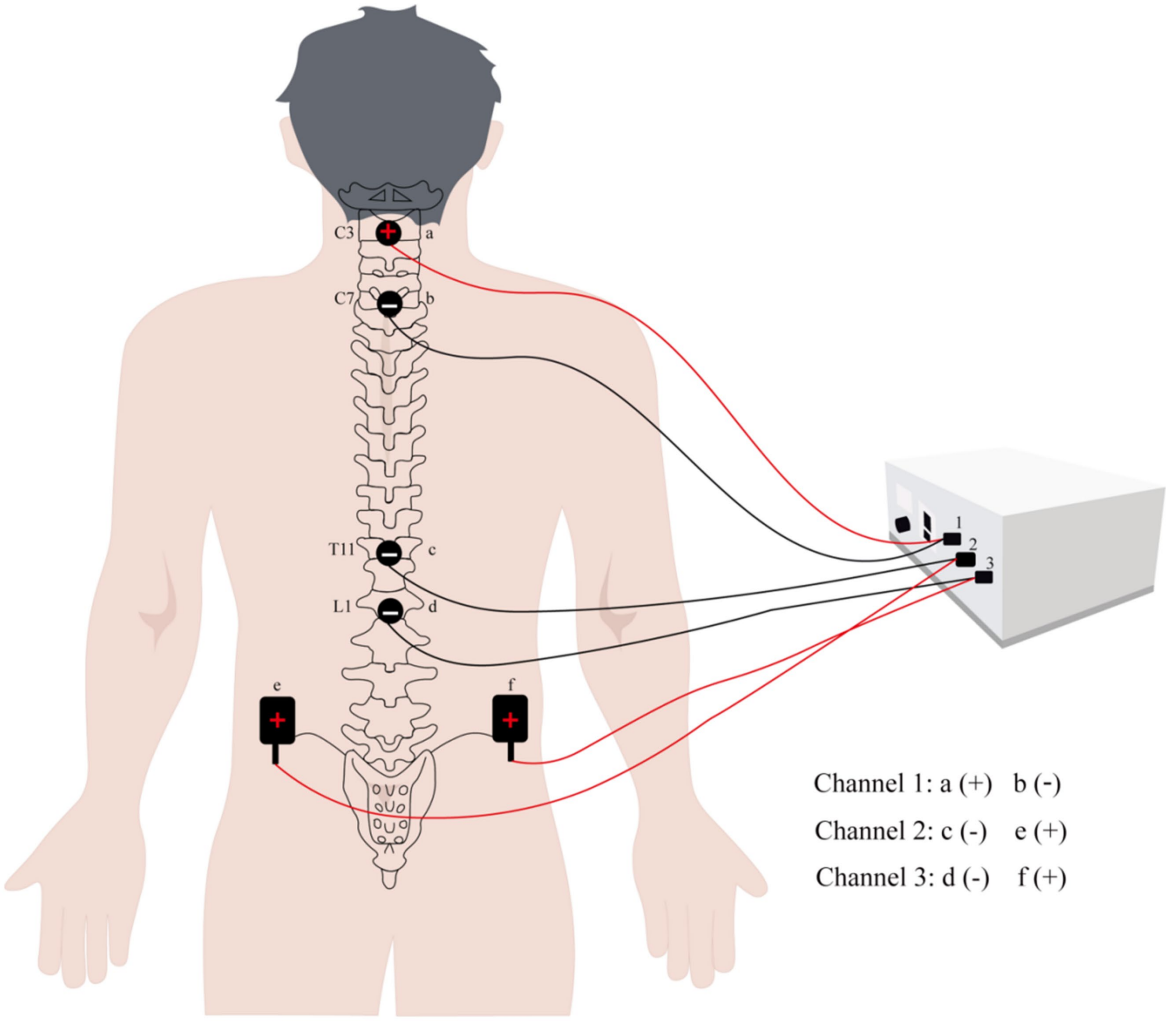
Schematic diagram of electrode sheet position and electrode connection for tSCS.

The electrode (5 × 5 cm, Hwato SDZ-II, Suzhou Medical Supplies Factory Co, China) sheets were applied onto the patient’s body and linked to an electro stimulator (KD-2A, transcutaneous electrical nerve stimulator (TENS), Beijing Yao Yang Kang Da Medical Instrument Co, China). The electrodes were placed on C3–C7, T11-L1 spine and anterior superior iliac spine. The stimulation waveform used biphasic, rectangular, 0.2 ms pulses at a frequency of 30 Hz, and the stimulation time was 30 min. The stimulation intensity was suitable for the patient to feel the microcurrent and no pain (60–80 mA) (Martin, 2021) (Figure 3).

Both Virtual Reality-based arm and leg cycling and tSCS were done simultaneously. The treatment was carried out 30 min at a time, once a day, 5 days a week and for 6-weeks (Supplementary Table S2).

### 3.3 Outcome

The findings of the 6-week duration are outlined in this report, indicating a notable enhancement in the patient’s state following the intervention.

**Figure 4:**
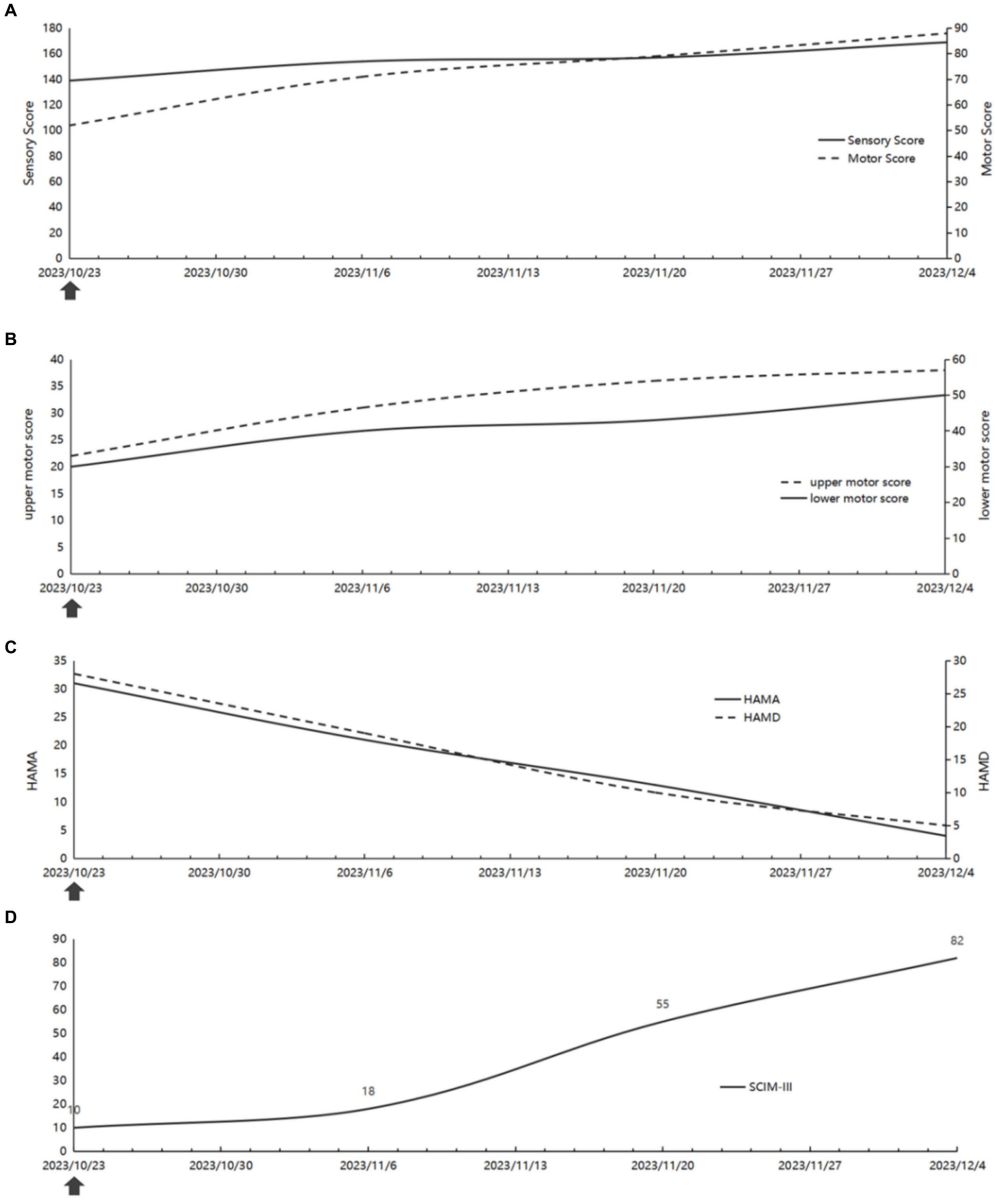
The changes of the patient’s function. **(A)** The ASIA score **(B)** The upper and lower motor score. **(C)** The HAMA and HAMD score. **(D)** The SCIM-III score. The arrows represent the start of virtual reality-based arm and leg cycling combined with transcutaneous electrical spinal cord stimulation after completion of the assessment.

The maximum muscle force of the primary muscles in the upper and lower limbs of the patient varied between 3 and 5 levels by the manual muscle testing. It is worth mentioning that the muscle strength of the right middle finger flexor and little finger abduction ranged from 0 to 3 levels. Subsequently, a comparative analysis was conducted on the ASIA scale scores between October 23, 2023, and December 04, 2023. The examination revealed a notable increase in motor scores, rising from 52 to 88, alongside a corresponding elevation in sensory scores, ascending from 139 to 169 (Figure 4A). The findings from the comparison of motor scores and sensory scores indicate a correlation with the rate of functional recovery. Our analysis involved the separate calculation of motor scores for the upper and lower extremities, revealing superior improvement in the lower extremities (Figure 4B).

The patients underwent administration of Hamilton anxiety (HAMA) and Hamilton depression (HAMD) tests at various stages of treatment to assess their psychological well-being. Notably, as somatic functioning improved, there was a significant reduction in anxiety and depression levels (Figure 4C).

In order to enhance activities of daily living (ADL), the patient initially exhibited limited proficiency and required substantial assistance to perform daily tasks. However, it is noteworthy that she has since made significant progress, transitioning from minimal mobility in bed to utilizing walking aids to independently traverse a distance of at least 300 m (Supplementary Figure S1A). Furthermore, a comparative analysis was performed on the SCIM-III scores during the period spanning from October 23, 2023 to December 04, 2023. The results obtained from the SCIM-III scale indicated a significant increase in scores, rising from 10 to 82, subsequent to the implementation of combination therapy (Figure 4D). Consequently, both the manifestations and the scores demonstrated an improvement in activities of daily living (ADL).

In light of this, we examined the MRI and observed an amelioration in the compression of the spinal cord (Supplementary Figure S1B). The severity of compression, hemorrhage, and swelling of the spinal cord is directly associated with the prognosis of neurological recovery (Miyanji et al., 2007). Traumatic spinal cord injuries are often followed by severe spinal cord compression, hemorrhage, and swelling, resulting in severe neurological deficits (Hamamoto et al., 2007; Skeers et al., 2018). Decompression surgery can relieve spinal cord compression, but swelling remains after the injury. It has been shown that compared to natural recovery, exercise reduces the area of the injured spinal cord cavity in spinal cord-injured rats, decreases the degree of inflammatory response and swelling, and promotes the expression of brain-derived neurotrophic factor, which in turn improves functional deficits (Park et al., 2020). Furthermore, additional research has demonstrated that implementing exercise interventions earlier can result in a more significant reduction in the size of the injury core, thereby aiding in the mitigation of inflammatory responses and the reduction of swelling (Brown et al., 2011). Inflammatory response and swelling are reduced after spinal cord injury, but according to the above study, the intervention of exercise and other combined therapies is more effective in reducing the inflammatory response and swelling as well as facilitating functional improvement after spinal cord injury as compared to natural recovery.

## 4 Discussion and conclusion

The case report involved a discussion on the physical therapy methods and clinical reasoning employed for a patient with traumatic SCI. Based on the clinical examination, a novel intervention approach was implemented, combining Virtual Reality-based arm and leg cycling with tSCS. This intervention approach specifically targeted the early treatment of patients with cervical SCI. Previous studies have shown that tSCS or Virtual Reality-based rehabilitation has effectively enhanced the function of individuals with SCI (Scandola et al., 2020; Barss et al., 2022). However, in contrast to these approaches, we have opted for a combined therapy that takes into consideration the anatomical structures involved, aiming to achieve comprehensive intervention by targeting the brain, spinal cord, and limbs.

In our study, improvements in the treatment of sensory and motor function were achieved through the implementation of Virtual Reality-based arm and leg cycling, which is a form of Virtual Reality-based rehabilitation. This treatment approach involves a well-defined mechanism of arm and leg cycling, which serves to enhance the coupling between the cervical and lumbar regions. This coupling is facilitated by the fasciculus proprius, a structural connection within the spinal cord. A study demonstrated that arm and leg cycling can effectively modulate spinal reflexes, thereby promoting motor function by strengthening the cervicolumbar coupling (Zhou et al., 2018). Further studies have provided evidence that cycling, specifically the movement linked to somatosensory response, can promote the regeneration of axons in propriospinal interneurons. Additionally, these interneurons have the ability to integrate into the existing locomotor circuitry within a controlled animal model of complete SCI (Flynn et al., 2011; Vasudevan et al., 2021). However, it is worth noting that Virtual Reality technology has the potential to compensate for the limited enjoyment and interactive nature of arm and leg rides, thereby enhancing their overall effectiveness. Moreover, it is crucial to emphasize the significance of early intervention in augmenting multisensory body representations and reinstating cortical control over leg movements in individuals with SCI (Mac-Thiong et al., 2021). Consequently, the early exposure to task-specific experiences has been found to expedite the recovery process, thereby potentially offering significant implications for early rehabilitation following SCI (Galea et al., 2015; Daunoraviciene et al., 2018).

Based on the findings, electrical epidural stimulation (EES) represents an emerging neuromodulation approach wherein an internal device is surgically implanted (Wagner et al., 2018). This method facilitates the recovery of voluntary muscle control and the restoration of motor function among patients. However, it is important to note that EES is associated with considerable surgical risks, substantial financial costs, vulnerability to post-surgical infections, and the potential displacement of electrodes. As a result of the limitations observed in EES, we utilized tSCS as a means to restore supraspinal control over motor function by stimulating the afferent nerves of the dorsal root and promoting the formation of new synaptic connections between spinal interneurons and motor neurons (Atkinson et al., 2022). Previous study have demonstrated that tSCS significantly enhances corticospinal excitability compared to sham stimulation (Powell et al., 2018). Cervicolumbar coupling in multisite tSCS has been shown to enable neuromodulation across various segments of the spinal cord (Samejima et al., 2022a,b). Furthermore, Muscle evoked potentials (MEPs), as an electrophysiological response that stimulates cortical areas of the brain to produce electrical stimuli that cause muscle myofibers to contract, are a valid assessment tool after spinal cord injury. A study involving animal subjects has provided evidence that incorporating anatomical current-flow modeling and physiological validation through MEPs can serve as a framework for enhancing tSCS interventions (Williams et al., 2022). By targeting representative groups of motor pools, tSCS allows for neuromodulation of corticospinal motor drive that is specific to both muscle and response (Minassian et al., 2016). In order to optimize motor function in the upper and lower extremities, the tSCS technique employs stimulation of the dorsal roots of cervical and lumbosacral bulging nerves, which govern movement in these respective areas (Atkinson et al., 2022). This neuromodulation approach encompasses various segments of the spinal cord. In summary, we employed a combination of Virtual Reality-based arm and leg cycling, along with tSCS, to synchronize the movements of the limbs through both physical and electrical stimulation. Our focus was on the anatomical linkage between the cervical and lumbar regions. The objective of this approach is to augment overall functionality by engaging the brain, spinal cord, and peripheral systems.

In this case report, we present preliminary research regarding the potential effectiveness of integrating Virtual Reality-based arm and leg cycling with tSCS. Nonetheless, additional investigation through randomized clinical trials is imperative to ascertain the clinical impacts. It is important to note that the patient was transferred to the rehabilitation department and received Virtual Reality-based arm and leg cycling combined with tSCS, thereby maximizing the recovery period and augmenting the therapeutic outcome in conjunction with conventional rehabilitation. After a mere 6-week period of intervention, the patient exhibited notable enhancements in somatic, psychological, and functional domains, suggesting that the utilization of Virtual Reality-based arm and leg cycling with tSCS may yield advantageous outcomes in said domains. Specifically, the patient’s motor function experienced significant improvement, as evidenced by improved ASIA scores in both upper and lower extremities post-treatment, with the most substantial improvement observed in the lower extremities. One potential explanation is that subsequent to cervical SCI, the motor nerves of the upper extremities demonstrate a heightened propensity toward the spinal cord center in contrast to the lower extremities (Oh et al., 2023). Consequently, the involvement of nerves in the upper limbs is more prominent than in the lower limbs. Therefore, in the methodology employed by this study, it was observed that the rehabilitation of the lower limbs exhibited greater efficacy, potentially attributable to the combined influence of motor feedback originating from the limbs, stimulation aimed at augmenting cortical activity, and the reinforcement of cervical-lumbar coupling through electrical stimulation at diverse spinal cord locations. These findings are consistent with analogous results reported in prior investigations.

This report possesses certain limitations. Firstly, the selection of each stimulation parameter for tSCS was based on the synthesis of various articles pertaining to tSCS treatment in patients with SCI. However, the investigation and validation of this stimulation parameter selection remain incomplete in the extant scholarly literature, thereby necessitating further examination. Additionally, the intervention duration for the patient in this particular case was 6 weeks. However, there may be persistent effects after 6 weeks (Pena et al., 2020). Finally, given that the current study is only a case report, it is not possible to know with certainty which intervention or natural recovery from removal of compression is most associated with positive change because there is no control. Therefore in order to determine the efficacy of Virtual Reality-based arm and leg cycling combined with tSCS in patients with early cervical SCI, a randomized controlled trial with a larger sample size must be conducted.

This case study provides a comprehensive account of the successful rehabilitation of a patient diagnosed with SCI. The therapeutic intervention involved the integration of Virtual Reality-based arm and leg cycling, in conjunction with transcutaneous electrical stimulation of the spinal cord. After a duration of 6 weeks, significant improvements were observed in sensory conduction, nerve functionality, and muscle strength, as well as substantial changes in the patient’s physical, cognitive, and functional domains.

## Data availability statement

The original contributions presented in the study are included in the article/Supplementary material, further inquiries can be directed to the corresponding authors.

## Ethics statement

The studies involving humans were approved by the Ethics Committee of Tianjin Hospital, Tianjin, China, under the ethical number 20222 Medical Lun Audit 209, and registered with the China Clinical Trial Registry under the registration number ChiCTR2300067335. The studies were conducted in accordance with the local legislation and institutional requirements. The participants provided their written informed consent to participate in this study. Written informed consent was obtained from the individual(s) for the publication of any potentially identifiable images or data included in this article.

## Author contributions

XC: Writing – original draft. SL: Writing – original draft. XZ: Writing – review & editing. TL: Writing – review & editing. ZX: Writing – review & editing. QinL: Writing – review & editing. QiL: Writing – review & editing.
