# Supplementary material for: Case report: Virtual reality-based arm and leg cycling combined with transcutaneous electrical spinal cord stimulation for early treatment of a cervical spinal cord injured patient
Source: Front Neurosci. 2024 May 17;18:1380467. doi: 10.3389/fnins.2024.1380467 (PMC11140104; doi:10.3389/fnins.2024.1380467)
Supplement: Supplementary file 1 [file Data_Sheet_1.docx]

Supplementary Material

**Case report:Virtual Reality-Based Arm and Leg Cycling Combined with Transcutaneous Electrical Spinal Cord Stimulation for Early Treatment of a Cervical Spinal Cord Injured Patient**

**Xiaolei Chu^1†^, Shuaiyi Liu^2†^, Xiaoxuan Zhao^2^, Tao Liu^2^, Zheng Xing^1^, Qingwen Li^2*^, Qi Li^1*^**

*** Correspondence:**

Qingwen Li
leeqw1101@163.com

Qi Li

Liqi_82@126.com

†These authors have contributed equally to this work and share first authorship

# Supplementary Figures


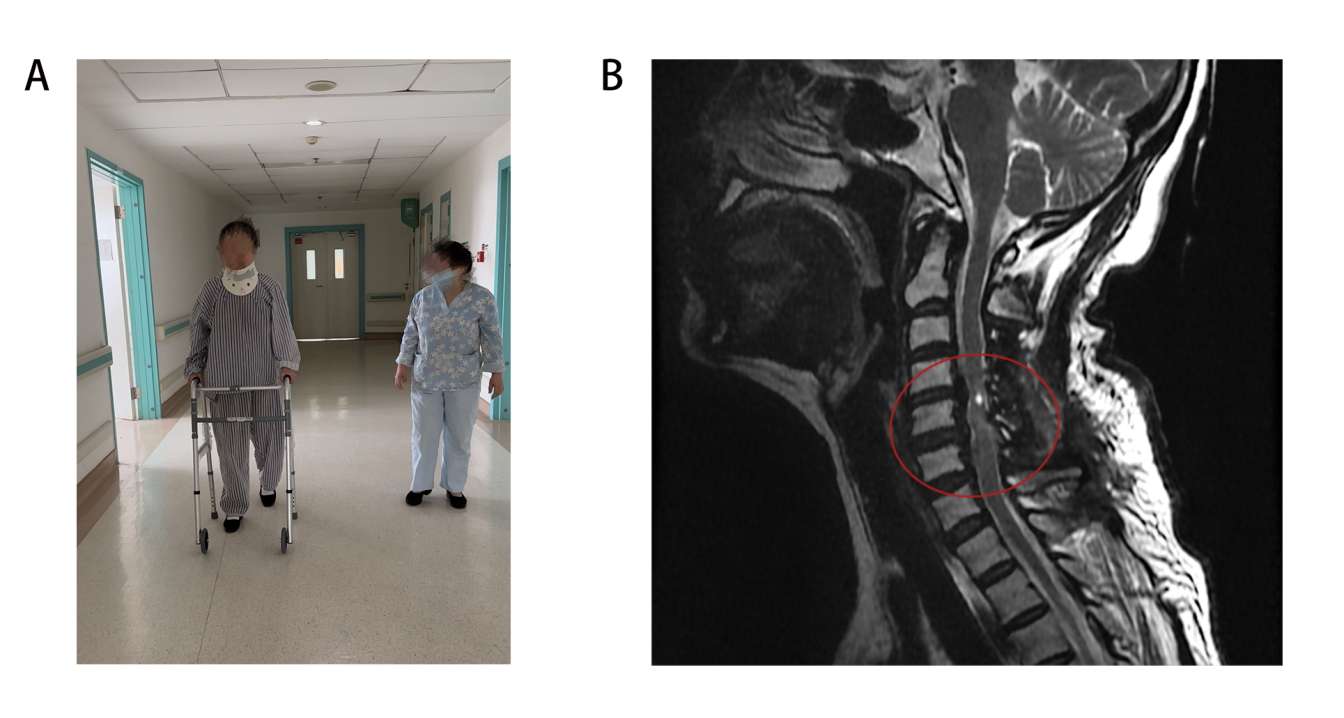


**Supplementary Figure 1.** The improvement of ADL. (A) Patient’s performance after combined treatment and rehabilitation: walk with walking aids. (B) Patient’s MRI after combined treatment and rehabilitation.

# Supplementary Tables

**Supplementary Table 1.** Assessment during bedside rehabilitation

| Date | ASIA | HAMA | HAMD | SCIM-III |
| --- | --- | --- | --- | --- |
| 2023/10/13 | 189 | 33 | 30 | 10 |
| 2023/10/23 | 191 | 31 | 27 | 10 |

**Supplementary Table 2.** Rehabilitation Training Program

|  | Routine Rehabilitation Training | Combination Therapies |
| --- | --- | --- |
| Program | Breathing Training | Virtual Reality-Based  Arm and Leg Cycling |
|  | Neuromuscular Electrical Stimulation | Transcutaneous Electrical  Spinal Cord Stimulation |
|  | Comprehensive Limb Training |  |
| Treatment frequency | 30 minutes for each treatment  once a day  five days a week  six weeks | |

Respiratory training is a training method to rebuild normal breathing pattern through various breathing exercises, which can prevent the occurrence of respiratory complications after spinal cord injury.

Neuromuscular electrical stimulation is a therapeutic method to prevent muscle atrophy by stimulating the muscles with low-frequency pulses.

Comprehensive limb training is a training method for patients' limb and trunk muscle strength, mobility and daily life ability.
